# Ultrastructural and Immunohistochemical Features of Telocytes in Placental Villi in Preeclampsia

**DOI:** 10.1038/s41598-018-21492-w

**Published:** 2018-02-22

**Authors:** Natalia V. Nizyaeva, Tatiana V. Sukhacheva, Roman A. Serov, Galina V. Kulikova, Marina N. Nagovitsyna, Natalia E. Kan, Victor L. Tyutyunnik, Stanislav V. Pavlovich, Rimma A. Poltavtseva, Ekaterina L. Yarotskaya, Aleksandr I. Shchegolev, Gennadiy T. Sukhikh

**Affiliations:** 1 National Medical Research Center for Obstetrics Gynecology and Perinatology, Moscow, 117997 Russia; 2 A.N. Bakulev National Medical Research Center of Cardiovascular Surgery, Moscow, 119991 Russia; 3 I.M. Sechenov First Moscow State Medical University, Moscow, 121552 Russia

## Abstract

A new cell type, interstitial Cajal-like cell (ICLC), was recently described in different organs. The name was recently changed to telocytes (TCs), and their typical thin, long processes have been named telopodes (Tp). TCs regulate the contractile activity of smooth muscle cells and play a role in regulating vessel contractions. Although the placenta is not an innervated organ, we believe that TCs are present in the placenta. We studied placenta samples from physiological pregnancies and in different variants of preeclampsia (PE). We examined these samples using light microscopy of semi-thin sections, transmission electron microscopy, and immunohistochemistry. Immunohistochemical examination was performed with primary antibodies to CD34, CD117, SMA, and vimentin, and TMEM16a (DOG-1), the latter was used for the diagnosis of gastrointestinal stromal tumours (GIST) consisting of TCs. We have identified a heterogenetic population of ТСs in term placentas, as these cell types differed in their localization, immunophenotype and ultrastructural characteristics. We assume TMEM16a could be used as the marker for identification of TCs. In PE we have revealed telocyte-like cells with ultrastructural signs of fibrocytes (significant process thickening and the granular endoplasmic reticulum content was increased) and a loss of TMEM16a immunohistochemical staining.

## Introduction

Preeclampsia (PE) is a multisystem pathological condition with clinical manifestations appearing after the 20^th^ week of gestation. This condition is characterized by increased blood pressure >140/90 mm Hg, oedema and proteinuria >0.3 g/day. In accordance with when the symptoms occur, PE is defined as early-onset PE and late-onset PE (with the former and the latter occurring before and after 34^th^ weeks of gestation, respectively)^1^. During early human pregnancy, extravillous cytotrophoblasts invade the wall of the uterus, and spiral arteries transform them into large vessels of low resistance. Failed trophoblast invasion and spiral artery transformation leading to hypoxia occur in preeclampsia, restricting foetal growth^2,3^. Placental blood vessels are not innervated, and regulation of their vascular tonus is controlled by molecules brought by the blood supply^4^. However, vasoconstriction of the placental villous tree may also be very important in PE^4^. Recent data show that cells with pacemaker activity, named telocytes (TCs), promote smooth muscle cell contractions by generating electrical pulses^5–10^, suggesting that they could be potential regulators of vascular tonus in placental villi.

To date, limited studies have observed TCs in the walls of villi vessels in physiological pregnancy and obstetric pathologies^11–14^.

The aim of the study was to study the ultrastructural and immunohistochemical characteristics of telocytes from placental villi in early-onset and late-onset preeclampsia.

## Results

### Control groups

The histological examination of placenta sections stained with haematoxylin and eosin from the physiological full-term pregnancies showed normally capillarized villous trees with a balance of mature intermediate and terminal villi. Immature intermediate villi were present as small accumulations. All stem villi were completely formed.

**Figure 1:**
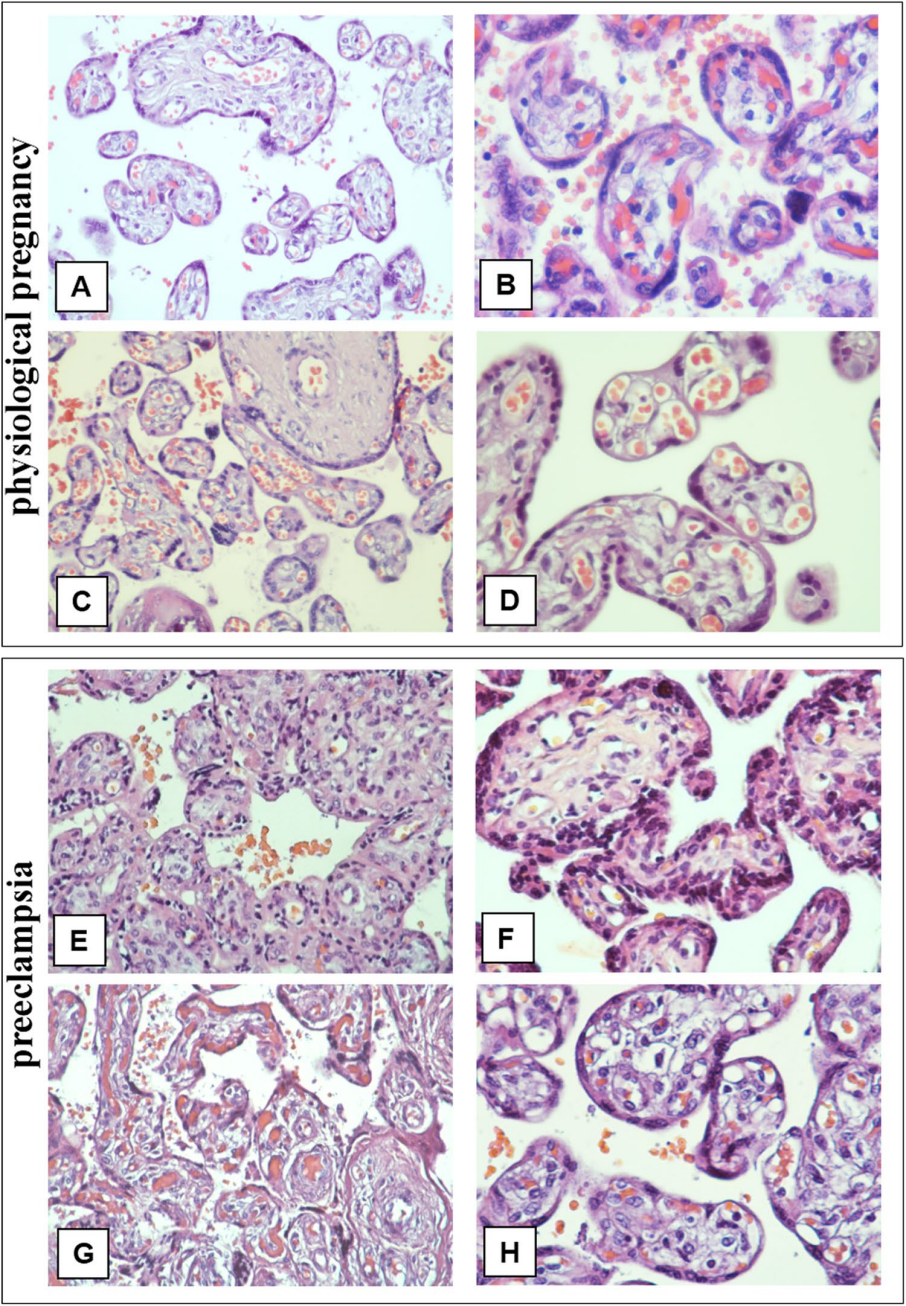
Histological examination of placental villous trees in preeclampsia and physiological pregnancies (staining with haematoxylin and eosin). (**A–D**) Morphological changes in placental villous trees during physiological pregnancy. (**А** and **B**) Placenta at the 27^th^ week (intermediate villi dominate). (**A**) х200. (**B**) x400. (**C** and **D**) Placenta at the 38^th^ week (stem, intermediate and terminal villi were found). (**C**) х200. (**D**) х400. (**E** and **F**) Morphological changes in the villous trees in early-onset PE (at the 26^th^ week) (stromal fibrosis and fibrinoids are detected). (**E**) х200. (**F**) x400. (**G**) The stem villi depicted show a high degree of apoptosis in structural constituents. Hofbauer cells located inside the stromal channels are marked to differentiate them from other cell types. (**G**) х200. (**H**) Morphological changes in late-onset PE (at the 38^th^ week) (stem villi are not completely formed and stromal channels with circulating macrophages (Hofbauer cells) are present in the villi). (**H**) x400.

Histological examination of placenta sections from the control group at 26–31 weeks of gestation revealed villous trees with mature and immature villi (Fig. 1A–D). Dystrophic changes, signs of inflammation or circulatory disturbances were not detected in placental samples in the control groups.

**Figure 2:**
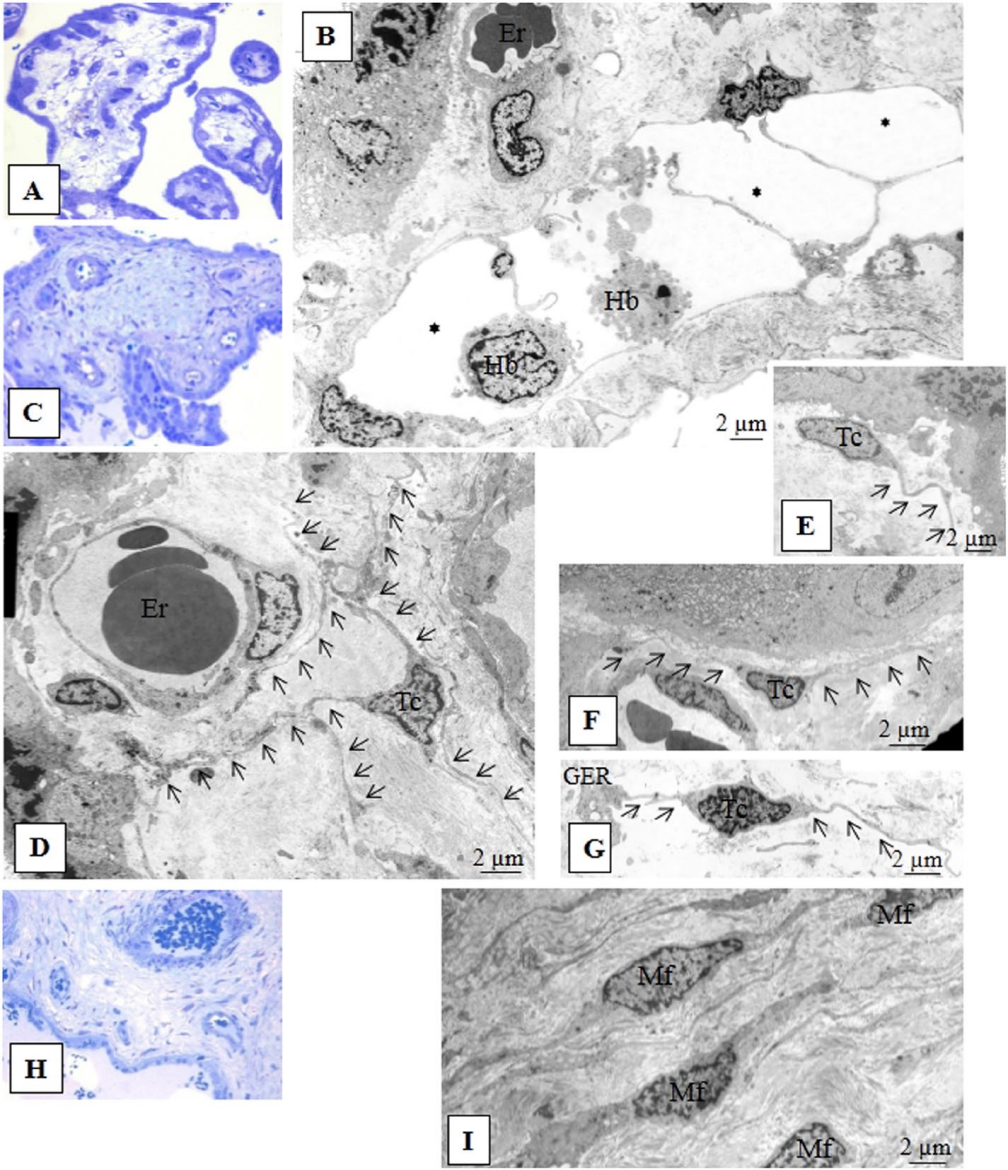
Morphology of villous stroma during physiological pregnancy. (**A**) Immature intermediate villi (capillaries are located peripherally and stromal channels in the centre). Semithin section, ×400 (Methylene blue staining). (**B**) Stromal channels formed by processes (telopodes) from several telocytes (average cell diameter 2.85 ± 0.6 μm) are in the centre of the immature intermediate villus. Several thin, long telopodes (average diameter 0.23 ± 0.08 μm) that are practically free of organelles contact one another and form a network of stromal channels in the lumen where macrophages (Hofbauer cells (Hb) are located. (Stromal channels are labelled with asterisks) White blood cells migrate from neighbouring blood vessels to the connective tissue to fulfil its macrophage functions, which is shown. A small number of granular endoplasmic reticulum cisternae were detected in the telopode dilatations only. Electron microphotograph at ×1400. (**С**) Mature intermediate villus stroma detect collagen deposits. The stromal channels are absent and blood vessels are formed. Semithin section, ×400 (Methylene blue staining). (**D**) In the stroma of a mature intermediate villus with collagen deposits, there are stellate telocytes (average diameter 2.96 ± 0.8 μm) with 3–4 telopodes (average diameter 0.23 ± 0.1 μm) forming a network around the blood vessels. Telopode dilatations reach more than 1.16 μm. Electron microphotograph, ×4800. Processes of telocytes are marked with aarrows. Erythrocytes are in the lumen of the vessel (Er). (**E**) In the mature intermediate villus stroma below the basement membrane of cytotrophoblast, spindle-shaped telocytes (Tc) are present (a mean diameter of 2.65 ± 0.9 μm) with oblong nuclei and usually 2 thin telopodes (average diameter 0.29 ± 0,1 μm). The extended processes from these telocytes form a chain under the basement membrane. Smooth and granular endoplasmic reticulum (GER) and mitochondria are mostly located in the telopode dilatations. Electron microphotograph, ×4800. (**F**) In some fields, in the stroma of mature intermediate villi, telocytes are observed under the basement membrane of trophoblasts. Spindle/stellate cells form and more than 2 processes make contacts with the stellate telocytes located deeper. Electron microphotograph, ×4800. (**G**) Cisternae of granular endoplasmic reticulum (GER), single mitochondria, and glycogen granules located in the perinuclear zone and in telopode dilatations. Mature intermediate villus stroma. Electron microphotography, ×5600. Processes of telocytes are marked with arrows. (**H**) Stem villus. Semithin section, ×400. (**I**) In the stroma of stem villi myofibroblasts (Mf) (average diameter 2,98 ± 1,1 μm) with 2 prolongations (average diameter 0,23-1,16 μm) in the arterial adventitia form a network in smooth muscle wall of blood vessels. Cisternae of well-developed granular endoplasmic reticulum locate near nucleus and in processes, small number of myofibrillae together with dense bodies reveals at the periphery of the cell (characteristics of both fibroblasts and smooth muscle cells). Electron microphotograph, ×5600.

Electron microscopy of placenta samples from the patients with physiological pregnancy displayed several types of TCs in the villi stroma. TCs were 2.85 ± 0.6 µm in diameter with 0.23 ± 0.08 µm-thick Tps (I type) (Fig. 2A and B) and were found in immature intermediate villi. The cells‘ nuclei were surrounded by a thin cytoplasmic layer, and a few granular endoplasmic reticulum cisternae were revealed in local Tp dilatations. TCs form a network via Tps and delimit stromal channels, where Hofbauer cells are located.

These cells were previously defined as reticular cells or foetal fibroblasts^4,15–17^. However, considering their morphological characteristics (long thin processes with which the cells interact and form a network), these cells may be classified as TCs^18^. The formation of a network via Tps is one of the key characteristics that allow these cells to be defined as TCs^13–15,19^.

One population displayed spindle-shaped cells (type II TCs) located under the syncytiotrophoblast basement membrane. These cells were connected with processes and formed a continuous chain under the trophoblast basement membrane. Type II TCs contain elongated nuclei surrounded by a thin cytoplasmic rim (Fig. 2E–G). Granular endoplasmic reticulum cisterns and other organelles were located around the nucleus and in local varicosities of the processes. Another TC population was characterized by stellate cells (type III TCs) (Fig. 2C and D) and was located deeper in the villous stroma. These cells had a greater number of processes than the spindle-shaped cells and formed a network around the villous stroma capillaries. The nuclei of both types of TCs were surrounded by a thin cytoplasmic layer. Smooth and rough endoplasmic reticulum cisternae, mitochondria and glycogen granules were revealed in telopode local dilatations (Fig. 2G and Table 1).

**Table 1:** Comparison of the ultrastructural features of mesenchyme cells in the.

Cell type	Localization	Form of cell / nucleus	Diameter of cell, µm; *m* ± *SD*	Diameter of nucleus, µm; *m* ± *SD*	Number of processes	Thickness of process, µm; *m* ± *SD*	Diameter of process dilatations, µm	Organelles
**Control group (physiological pregnancy)**								
TC I	In stroma of immature intermediate villi, forming stromal channels	Polygonal / irregularly- shaped nuclei	2.85 ± 0.6	2.24 ± 0.5	3–6	0.23 ± 0.08	1.69 ± 1.05	Small numbers of granular endoplasmic reticulum cistern were detected only in the telopode dilatations
TC II	In stroma of mature intermediate villi, under the trophoblast basement membrane	Spindle / oval-shaped nuclei with 1 nucleolus	2.65 ± 0.9	2.11 ± 0.8	2	0.29 ± 0.1	1.08 ± 0.2	Smooth and granular endoplasmic reticulum and mitochondria, mainly located in the telopode dilatations
TC III	In stroma of mature intermediate villi, forming a network around big vessels	Stellate / irregularly- shaped nuclei with 1–2 nucleoli	2.96 ± 0.8	2.22 ± 0.7	3–4	0.23 ± 0.1	1.12 ± 0.4	Granular endoplasmic reticulum cisternae, a single mitochondria, glycogen and granules located in the perinuclear zone and in the telopode dilatations
Myofibroblasts	In stroma of stem villi, forming a network in smooth muscle wall of blood vessels	Stellate and spindle / irregularly-shaped nuclei with 1-2 nucleoli	2.98 ± 1.1	2.17 ± 1.4	2–3	0.23 ± 1.16	more than 1.16	Well-developed granular endoplasmic reticulum cisternae located near the nucleus and in telopodes; small number of myofibrillae, together with dense bodies, revealed at the periphery of the cell (characteristics of both fibroblasts and smooth muscle cells)
**Preeclampsia**								
Cell types	Localization	Form of cell / nucleus	Diameter of cell, µm; *m *±* SD*	Diameter of nucleus, µm; *m *±* SD*	Number of processes	Thickness of process, µm; *m *±* SD*	Diameter of process dilatations, µm	Organelles
Polygonal telocyte-like cells	In stroma of immature intermediate villi	Polygonal / many-lobed nuclei with small lumps of heterochromatin on their periphery	3.25 ± 0.6	2.64 ± 0.7	3–6	0.28 ± 0.1	1.53 ± 0.6	Long thin processes surround the strom They display dystrophic changes and some are broken. A few granular endoplasmic reticulum cisternae, a single mitochondria, and smooth endoplasmic reticulum vesicles are detected in the perinuclear zone and in telopode dilatations.al channels.
Spindle-shaped telocyte-like cells	In stroma of mature intermediate villi, under the trophoblast basement membrane	Spindle-shaped form / oval-shaped nucleus with the heterochromatin lamps localized mainly along the periphery of the nucleus	1.92 ± 0.9	1.61 ± 0.8	2	0.22 ± 0.2	0.95 ± 0.4	The cell usually contains two processes extending in opposite directions and making contacts with neighbouring processes. The cells are injured and display dystrophic changes; some are broken, and the others have thickened. Several granular endoplasmic reticulum cisterns, smooth endoplasmic reticulum vesicles, single granules of glycogen and mitochondria are located in the perinuclear regions along the two poles of the nucleus and near the process extensions.
Stellate-shaped telocyte-like cells	In stroma of mature intermediate villi, forming a network around blood vessels	Stellate / Polygonal nucleus, with heterochromatin located along the periphery of the nucleus and small clumps of heterochromatin in the centre of it	2.90 ± 0.8	2.39 ± 0.8	3–4	0.21 ± 0.1	1.1 ± 0.3	3-4 thin processes depart from the cell in different directions and make contacts with adjacent processes, forming a network. Processes are associated with injuries and dystrophic changes. Some are broken and others are thickened. Granular endoplasmic reticulum cisternae, a single mitochondria, and glycogen granules are shown in the perinuclear zone and in the telopode dilatations.
Myofibroblasts	In stroma of stem villi, forming a network in smooth muscle blood vessel walls	Elongated / irregularly-shaped nuclei with nuclear envelope invaginations	2.89 ± 1.1	2.23 ± 1.0	2–3	0.38 ± 0.3	0/85 ± 0.5	Heterochromatin is located along the periphery of the nuclear envelope and its invaginations. 2-3 processes depart from the poles of the cell. The cells are usually arranged in parallel rows. They have ultrastructural characteristics of fibrocytes and display dystrophic changes (e.g. glycogen granule metachomacy phenomenon - becoming pink after staining with methylene blue and lipid droplets).
Fibrocytes	In stroma of stem and intermediate villi	Oblong form/ oblong nuclei with multiple lumps of heterochromatin merged into large conglomerates	2.28 ± 0.6	1.65 ± 0.6	2	0.40 ± 0.3	0.69 ± 0.2	2 thin processes depart in opposite directions from the poles of the cell. The cells are arranged in parallel rows. Single granular endoplasmic reticulum cisterns and glycogen granules are located near the nucleus poles and in the process dilatations. Lipid droplets are revealed.

placental villi in the preeclampsia and control groups.

Concentrically located cells (the cell diameters were 2.98 ± 1.1 µm, and the telopode thicknesses were 0.23 ± 0.1 µm) had ultrastructural features of fibroblasts (cisterns of well-developed granular endoplasmic reticulum located in the perinuclear cytoplasm and in processes) and smooth muscle cells (myofibrils and dense bodies located at the cell periphery). These cells contained well-developed granular endoplasmic reticulum cisternae with thin fibrillae and so-called “dense bodies” located at the periphery of the cell^18^ (Table 1).

Myofibroblasts were also found in the adventitia of large vessels in stem villi and in the perivascular area in large mature intermediate villi. Spindle-shaped and stellated myofibroblasts (2.98 ± 1.10 μm in diameter) had 2-3 processes per irregular-shaped nuclei (2.17 ± 1.40 μm in diameter) with 1-2 nucleoli. Process widths varied from 0.23 to 1.16 μm. These cells had similar ultrastructural features, including long thin processes and a small number of organelles in the cytoplasm around the nuclei and local varicosities of the processes. Depending on cell location and the degree of villous fibrosis, the number of processes varied, their widths tended to increase, and cytoplasm was enriched with organelles. Depending on differentiation, the cells similar to TCs with long thin processes (in immature intermediate villi) were replaced with cells with ultrastructural signs similar to fibroblasts and myofibroblasts (in stem villi). Neither telocyte-like cells nor myofibroblasts were detected in the terminal villi.

**Figure 3:**
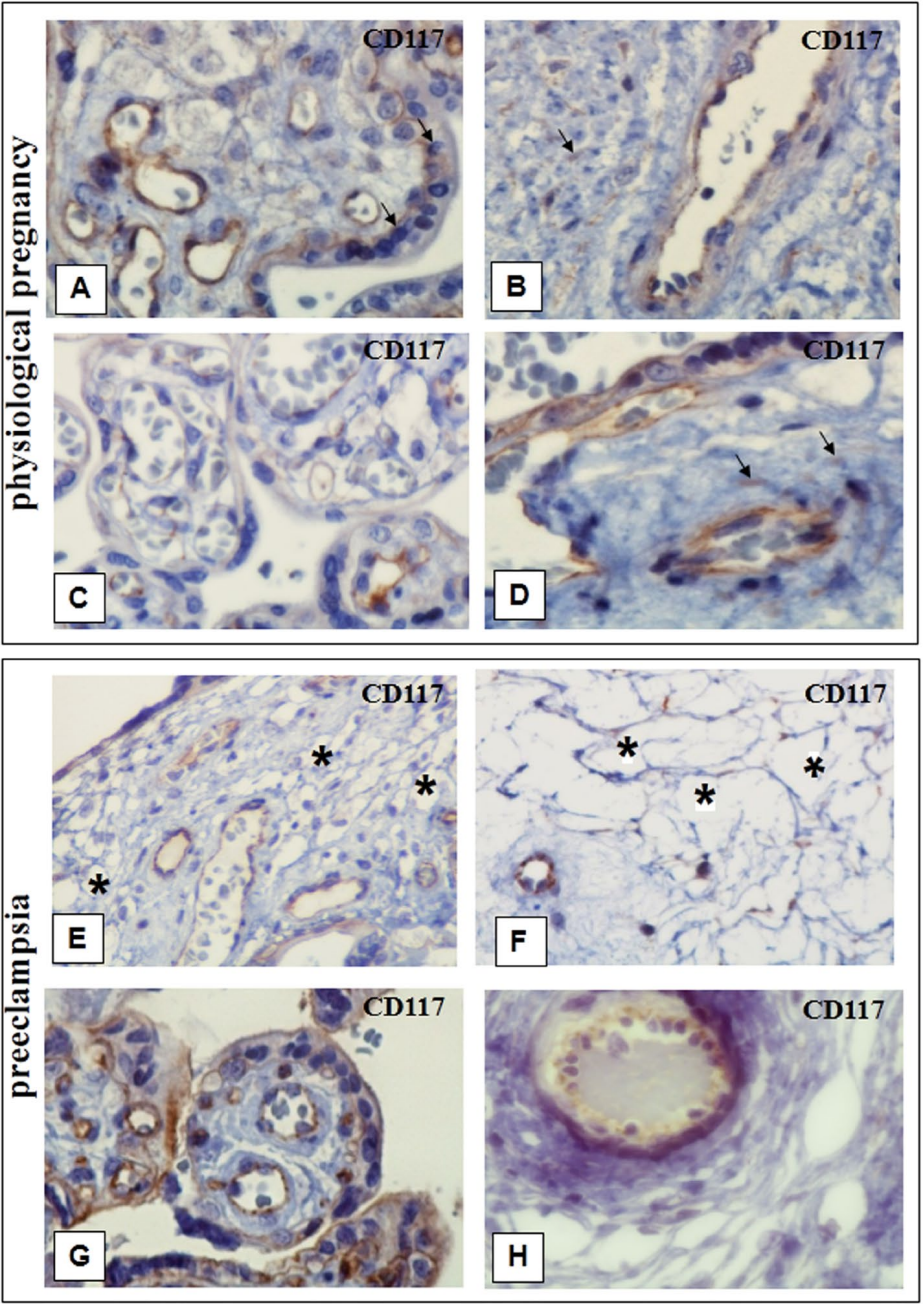
Immunohistochemical staining of placental villi with primary antibodies to CD117. in preeclampsia and physiological pregnancy. (**A–D**) Placental villous tree in a physiological pregnancy. (**А**) Intermediate villi (at the 27^th^ week). Weak staining of telocytes located under syncytiotrophoblast and deeper in the villi (only single cells) The telocytes located under the syncytiotrophoblast is weakly positive for the CD117 antibody, which is to be more intense in the blood vessel endothelium. (Processes of telocytes are marked with arrows), х400. (**B**) Stem villi (at the 27^th^ week). Weak staining of single myofibroblasts in the stem villus wall. The myofibroblasts are weakly positivity to the CD117 antibody. This signal is also weak in the blood vessel smooth muscle cells. However, the signal is more intense in the blood vessel endothelium. х600. (**С**) Intermediate villi (at the 39^th^ week): weak telocytes staining located under syncytiotrophoblast, and single telocytes deeper in villi), х600. (**D**) Stem villi (at the 39^th^ week). Positive staining of myofibroblasts in the wall, (Processes of telocytes are marked with arrows), х600. (**E** and **F**) Immature intermediate villi in early-onset PE (at the 27^th^ week). Staining of telocytes located under the syncytiotrophoblast and the staining of cells and their telopodes in the villus core (a large number of stromal channels formed by telocytes are present; the areas with disrupted connections between the cells and damaged telocytes and their telopodes are observed) (Stromal channels are marked with asterisks), (**E**) х400. (**F**) х600. (**G**) Intermediate villi in late-onset PE (at the 38^th^ week). Telocytes are only observed under the syncytiotrophoblast. There are single telocytes in the villous core. х400. CD117 expression observed in the endothelium and at the apex of the syncytiotrophoblast. In panel 3G, the stromal channels are negative to CD117, there is an intense positivity in the endothelium of large lumen blood vessels. (**H**) Stem villi in late-onset PE (at the 38^th^ week). The stromal channels remain (staining of the stroma from stem villi is negative). (**H**) х600.

**Figure 4:**
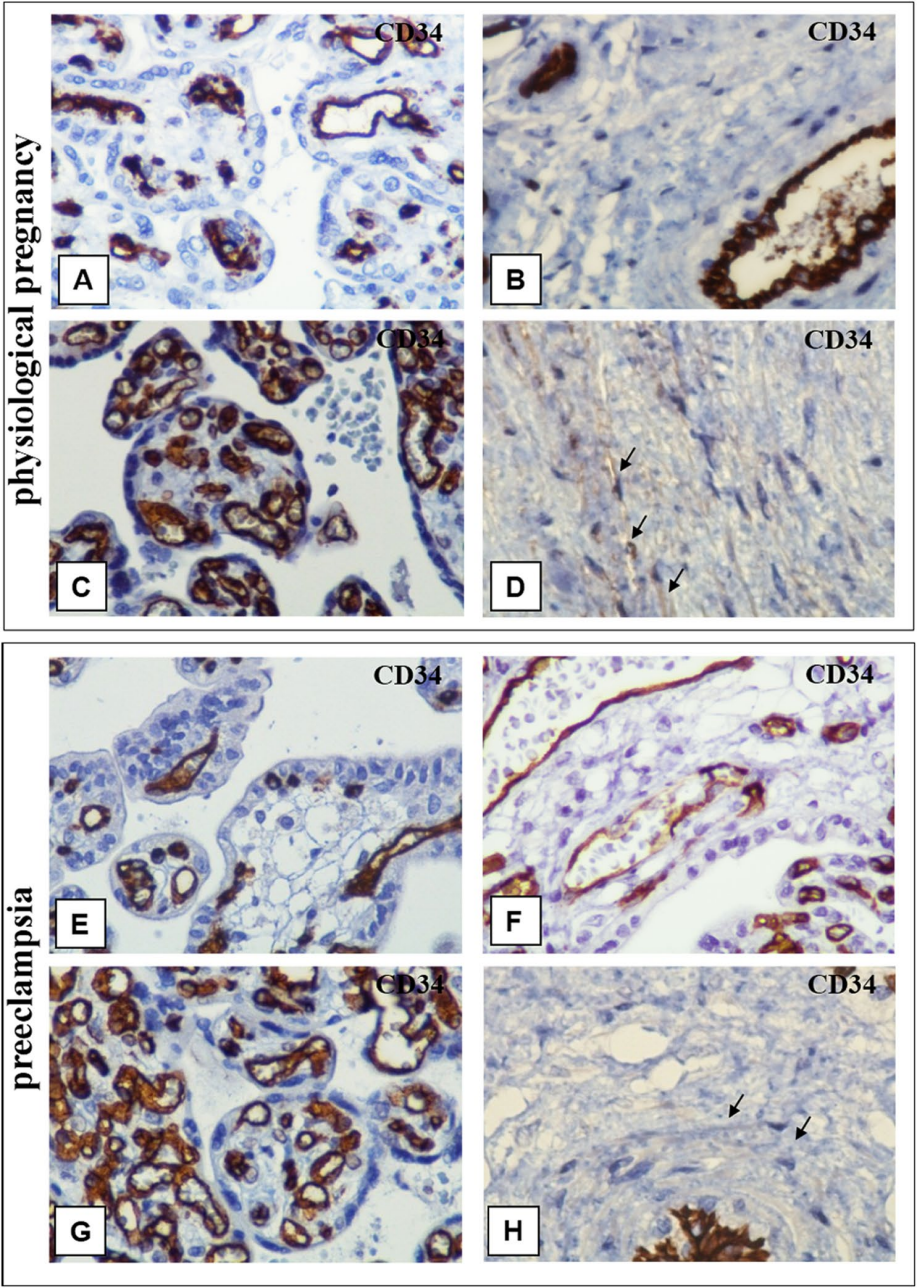
Immunohistochemical staining of placental villi with primary antibodies to CD34 in preeclampsia and physiological pregnancy. (**А–D**) Placental villous tree in a physiological pregnancy. (**А**) Placenta at the 27^th^ week (intermediate villi dominate), х400. (**B**) Stem villi at the 27^th^ week (the staining is not observed in the stroma), х600. (**С**) Intermediate villi at the 39^th^ week (only the staining of vascular endothelium is observed), х400. (**D**) Stem villi at the 39^th^ week (myofibroblasts are stained in the stem villus wall indicated with arrows), х600. (**E**) Intermediate placenta villi in early-onset PE (at the 27^th^ week) (a lot of stromal channels with circulating macrophages are shown in some intermediate villi; vascular endothelium staining is only observed; staining in not detected in the villous stroma), х400. (**F**) In early-onset PE (at the 33^th^ week), the stromal channels remain in the stem villi stroma (vascular endothelium staining is observed), х600. (**G**) Intermediate villi in late-onset PE (at the 38^th^ week) (only vascular endothelium staining is observed), х400. (**H**) Stem villi in late-onset PE (at the 38^th^ week) are not completely formed; single telocytes are stained weakly and the stromal channels are still present in them (indicated with arrows), х600.

Immature intermediate villi immunohistochemical analysis revealed CD117-positive staining (Fig. 3A,B) where TCs were located both under syncytiotrophoblasts and deeper in the villi. Only individual TCs deeper in the villi were positively stained with CD117 and CD34 (Fig. 4 and Тable 2). In mature intermediate villi, moderate CD117 (++) expression was localized under the syncytiotrophoblasts and weak irregular staining (+−) was deeper in the villi (Fig. 3C,D). Intense СD34 expression was detected in the villous vessel endothelium in all types. Moreover, staining in the villi stroma was predominantly negative (Fig. 4A–D), as only single СD34-positive cells were observed under the microscope in the fields of view (Fig. 4D and Table 2).

**Table 2:** Comparative immunohistochemical characteristics of telocytes from placental villi in patients in the preeclampsia and control groups.

Group	Control group				Patients with EPE				Patients with LPE			
	Immature inter-mediate villi	Mature inter-mediate villi		Stem villi (myofibroblasts)	Immature intermediate villi	Mature inter-mediate villi		Stem villi (myofibroblasts)	Immature inter-mediate villi	Mature inter-mediate villi		Stem villi (myofibroblasts)
Marker		under STB	in villous stroma			under STB	in villous stroma			under STB	in villous stroma	
Vimentin	++	++	+++	+++	++	++	++	++	++	++	+++	+++
CD34	−	−	+− *s. c*.	+	−	−	+− *s.c*.	−	−	+− *s.c*.	−	+− *s.c*.
CD117	+++*	++	+	+−++	+++*	+	+−	+−	+−*	+	+−	+−
SMA	−	−	−	+++	−	−	−	+−	−	−	−	+−++
TMEM16a (DOG-1)	+	+	+	++/+**	−	−	−	−	−	−	−	−

Comments.

*Note*: *–marked staining both in the villi stroma and under syncytiotrophoblasts.

**–With a gestation period of 26–31 weeks.

s.c - only in single cells.

Myofibroblasts in the stem villi were characterized by weak and irregular expression of CD117 and CD34, but they were stained strongly with vimentin (+++) and SMA (+++). They also showed weak and moderate immunohistochemical staining (+−++) for the telocyte-specific marker TMEM16a (DOG-1).

**Figure 5:**
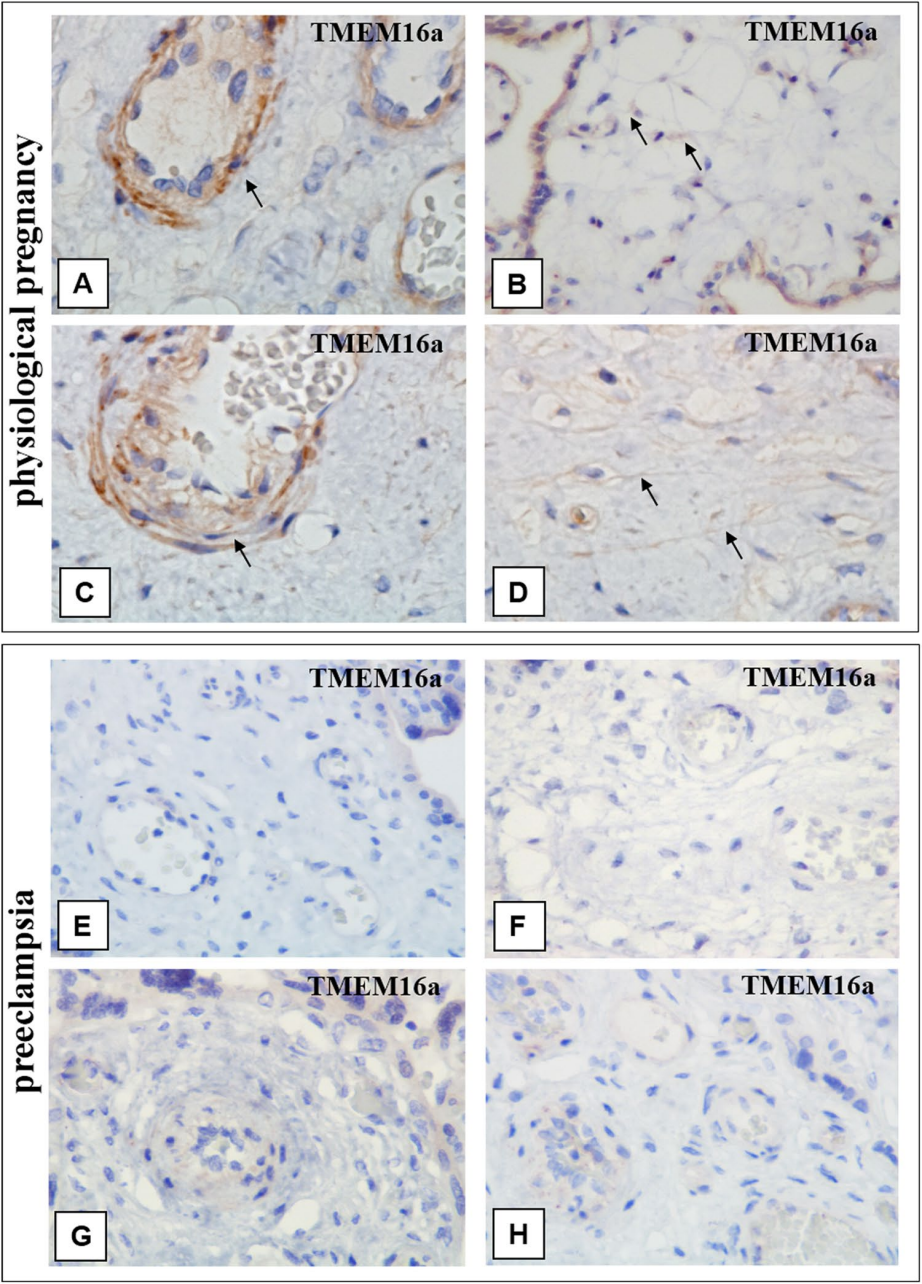
Immunohistochemical staining of placental villi with primary antibodies to TMEM16a in preeclampsia and physiological pregnancy. (**A–D**) Placental villous tree in a physiological pregnancy. (**A**) Weak staining of telocyte-like cells in the stem villi (at the 33th week). (**B**) Weak staining of telocytes in the stroma of immature intermediate villi. Telocytes delineate the vessel formation (at the 27^th^ week) (indicated with arrows). (**A**) x600. (**B**) x600. (**C** and **D**) Staining of (**C**) myofibroblasts in the vascular wall (at the 39^th^ week) and in (**D**) the stem villi stroma (telocytes and telopodes are indicated with arrows). (**С** and **D**) х600. (**E** and **F**) Villous trees in early-onset PE (at the 32th week). Staining of the stroma in the (**E**) intermediate villus and (**F**) stem villus is not shown. E and F. x600. G and H– Villous trees in late-onset PE (at the 38^th^ week). There is no staining of telocytes and myofibroblasts in the (**G**) intermediate villus and (**H**) stem villus. G and H. x600.

**Figure 6:**
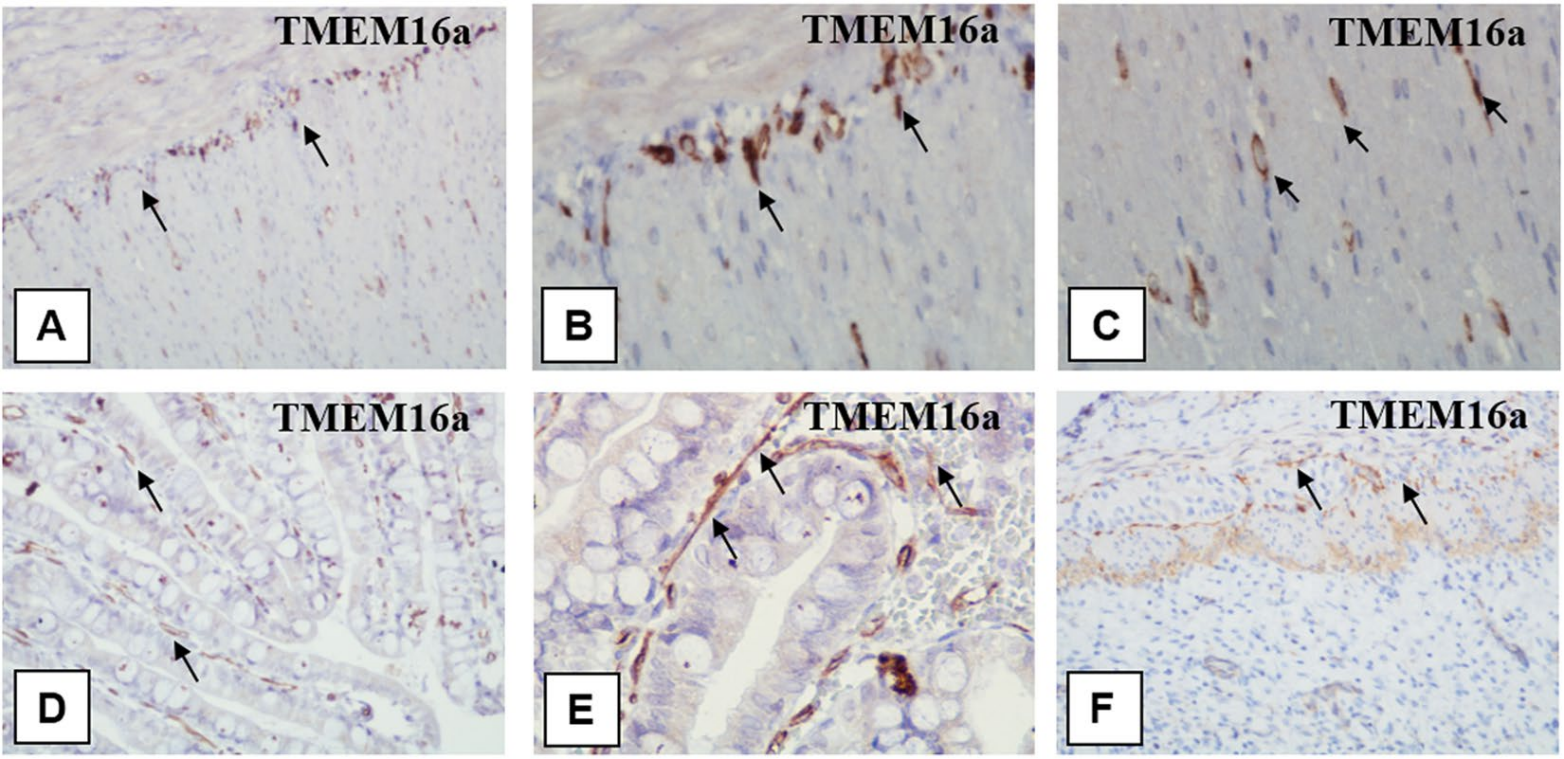
Immunohistochemical staining of the bowel wall with primary antibodies to TMEM 16a (*positive control*). (**А–Е**) The bowel wall (of a five-day-old newborn). Strong telocyte staining is detected in the smooth layer. Telocytes form enclosed structures and capillary walls in the same places. (**A**) х200. (**B**) x400. (**C**) x600. (**D**) and (**Е**) The presence of telocytes in the crypts and different layers (indicated with arrows). (**D**) х200. (**E**) x400. (**F**) The small intestine wall (of a foetus at the 20th week of gestation). Weak immunohistochemical staining of telocytes is detected in the muscle layer, x200.

TMEM16a expression is observed in TCs in all villi types (excluding terminal) in the control groups. However, more significant expression was detected in myofibroblasts in the vascular stem villi walls during full-term pregnancy (Fig. 5A–D and Table 2*)*. The TMEM16a positive control was intestine samples. Immunohistochemical study of the positive controls with primary antibodies to TMEM16a revealed moderate staining of TCs with long thin Tps that formed a network by interacting with one another (Fig. 6A–F).

### Preeclampsia

The histological examination results revealed that intermediate villi (mature and immature) with evident dystrophic changes and focal stromal fibrosis were prevalent in patients with early-onset preeclampsia. Only small stem villi were completely formed, while predominantly large stem villi were not.

Capillaries in intermediate villi were irregularly located and avascular villi were also present. Multiple villous tree infarctions were detected (Fig. 1E–H). Stem villi stroma were loose and soft in patients with LPE; occasionally, the persisting stromal channels were similar to honeycombs, and focally stromal channels persisted. Some intermediate villi contained collagen deposits, including fibrinoid deposits.

**Figure 7:**
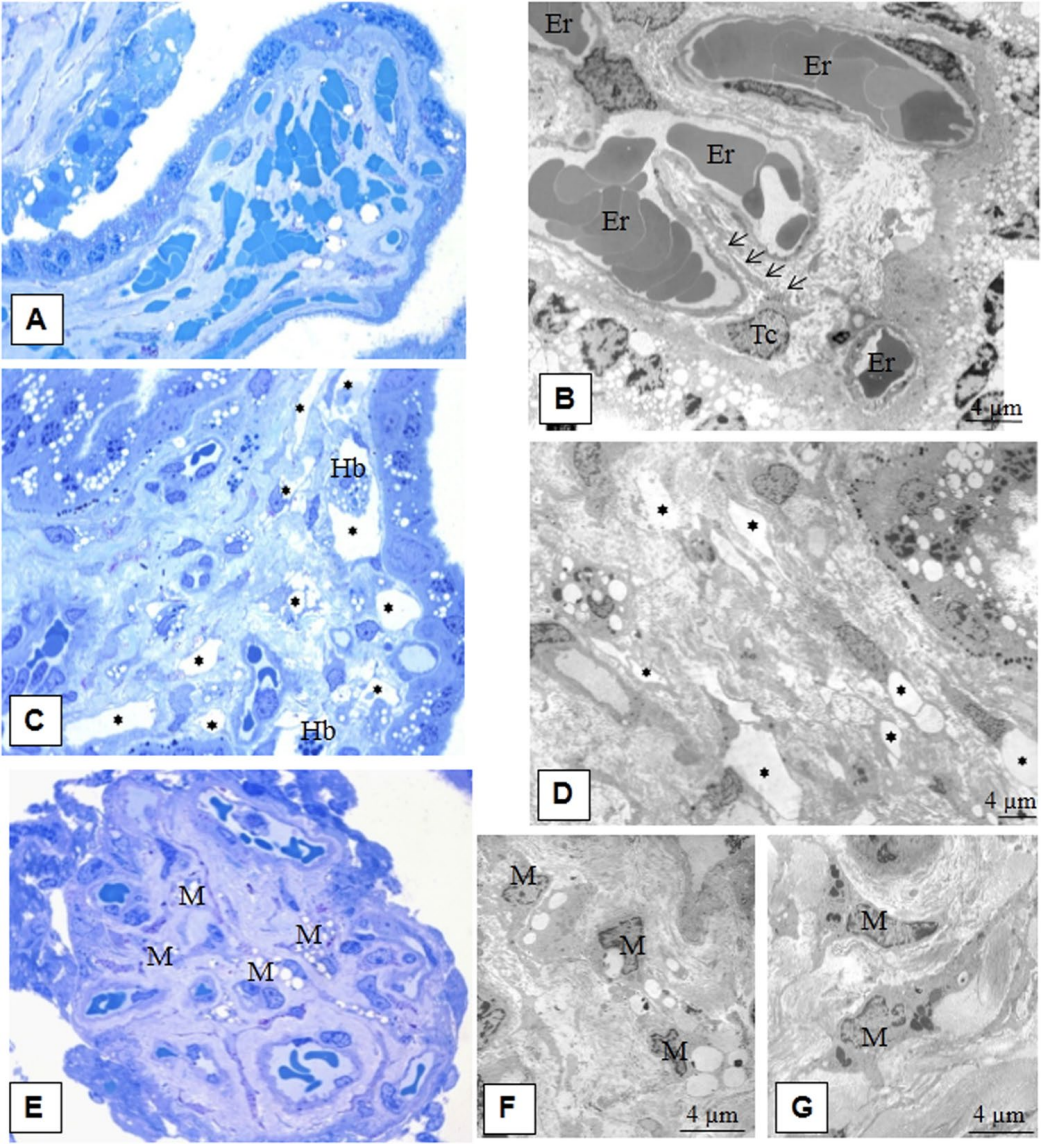
Morphology of the villous stroma of intermediate villi in preeclampsia. (**A**) Placental villi in preeclampsia. Sludged erythrocytes in the vascular lumen. Semithin section, ×400 (Methylene blue staining). (**В**) Stellate telocyte-like cells (Tc) are located near blood vessels. Slugged erythrocytes in the blood vessel lumen. The stroma contains collagen fibres and multiple vacuoles are seen in the syncytiotrophoblasts. Erythrocytes are in the lumen of the vessel (Er). Electron microphotograph, x4800. (**C**) Areas of telocyte-like cells form pseudo-stromal channels in mature intermediate villi. Semithin section, ×400 (Methylene blue staining) (areas with stromal channels are denoted with asterisks). (**D**) In the fibrotic villi stroma, telocyte-like cells (average diameter 3.25 0.6 μm) are seen due to their processes (diameter averages 0.28 ± 0.1 μm), which limit the pseudo-stromal channels. Formed vessels are shown nearby in stroma (areas with stromal channels denoted with asterisks). Electron microphotograph, ×1400. (**E**) Macrophages (M) with multi-lobed nuclei and multiple vacuoles. Semithin section, ×400. (**F**) Macrophages (M) with vacuoles. Electron microphotograph, ×4800. (**G**) Macrophages (M) with residual bodies. Electron microphotograph, ×4800.

The early- and late-onset PE analysis of semi-thin placental sections with methylene blue staining revealed fields of view with sludged erythrocytes in the blood vessels of placental intermediate villi (Fig. 7A,B). Clots and sludges were predominantly present in the lumen of capillaries in early-onset preeclampsia. Early onset preeclampsia capillaries showed different diameters and were irregularly located within the fibrotic stroma. Some villi in late-onset preeclampsia displayed completely formed capillaries containing stromal channels. Moreover, collagen deposits were detected in the villi stroma.

**Figure 8:**
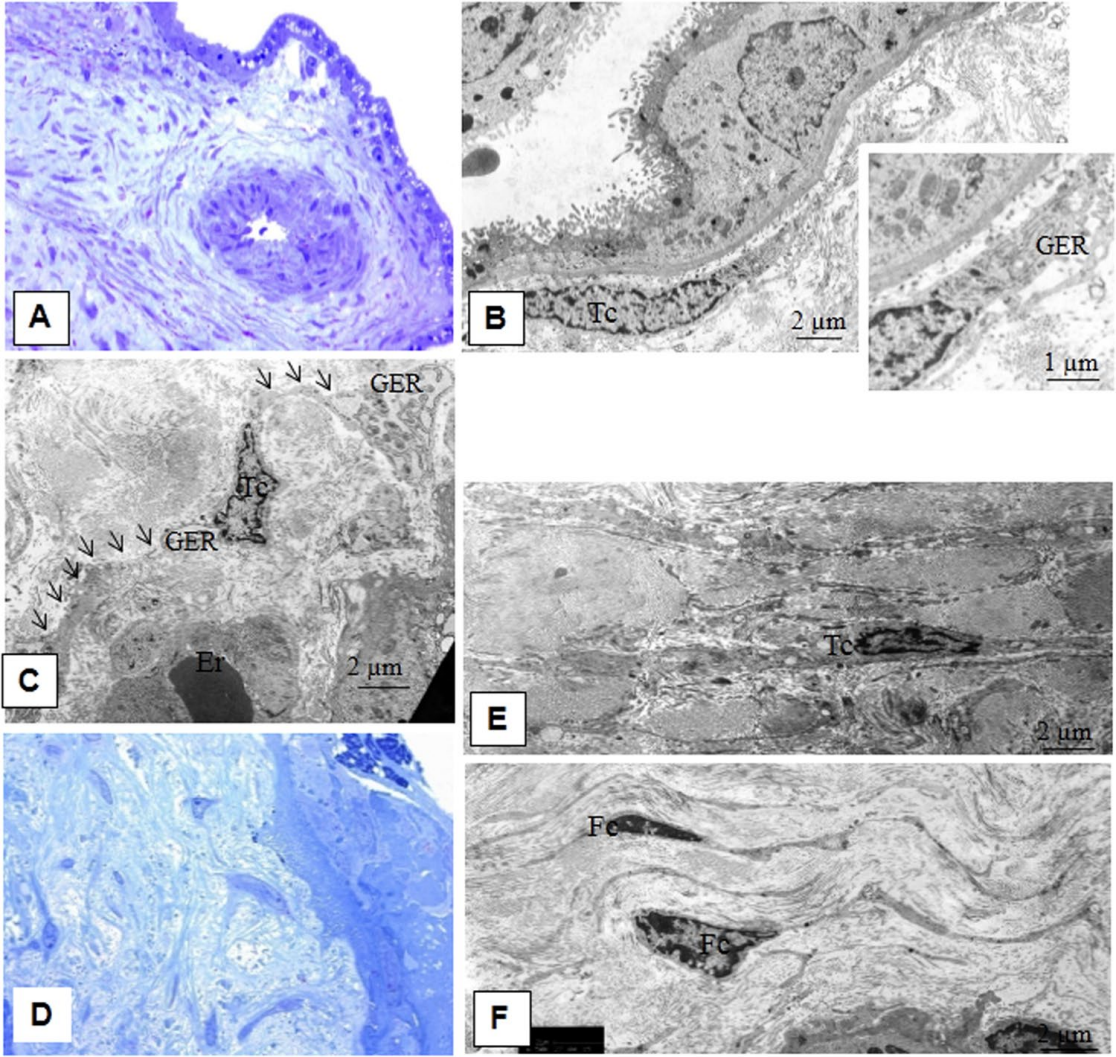
Morphological features of the stem villi stroma in preeclampsia. (**A**) Stem villi. Semithin section, ×400 (Methylene blue staining). (**B**) Spindle-shaped telocyte-like cells (Tc) (average diameter 1.92 ± 0.9 μm) are located under the syncytiotrophoblast basement membrane. Its processes (diameter a mean 0.22 ± 0.2 μm) form a network. In the perinuclear zone, these telocyte-like cells contain developed granular endoplasmic reticulum, indicating fibroblast differentiation (insert). Electron microphotographs, ×6500 and ×11000. (**C**) A stellate-shaped telocyte-like cell (Tc) (average diameter 2.89 ± 1.1 μm) with several processes (average diameter 0.38 ± 0.3 μm) is observed in the fibrous stroma of stem villi. Well-developed granular endoplasmic reticulum (GER) are revealed in the perinuclear zone and among process dilatations. Processes of telocyte-like cell are indicated with arrows. Electron microphotograph, ×6500. (**D**) Stem villus with advanced stromal fibrosis. Semithin section, ×400 (Methylene blue staining). (**E**) Stellate-shaped telocyte-like cells (Tc) with developed granular reticulum in the roughly fibrotic stroma. Electron microphotograph, ×6500. (**F**) Fibrocytes (Fc) (average diameter 2,28 ± 0.6 μm) with processes (average diameter 0.40 ± 0.3 μm) in a roughly fibrotic stroma. Fibrocyte nuclei with large heterochromatin lumps. There are single glycogen granules in the cytoplasm. Electron microphotograph, ×6500.

There were areas with stromal channels delimited by Tps near completely formed blood vessels (Fig. 7C,D). Histiocytes (macrophages) in the stroma of mature intermediate villi that contained numerous vacuoles and residual electron-dense bodies within their cytoplasm (Fig. 7E–G). The results from the ultrastructural analysis showed telocyte-like cells undergoing fibroblastic differentiation in early- and late-onset PE in the intermediate villi stroma. These cells had larger cytoplasmic volumes and an increased number of granular endoplasmic reticulum cisternae in their dilated perinuclear zone (Fig. 8A,B) and prolonged local dilatations (Fig. 8C and Table 1). Stromal fibrosis was observed in large mature intermediate villi in preeclampsia (Fig. 8D) with the gradual disappearance of endoplasmic reticulum cisternae from the telocyte cytoplasm. In this case, Tps were thicker (≥1.1 µm) than in the control group. Ultrastructurally, these cells were similar to fibrocytes **(**Fig. 8E,F).

Moreover, a significant range of cell sizes was observed (from 1.80 to 3.25 µm). This difference was associated with the fibroblast cell differentiation and with notable dystrophic changes (Table 2).

Similar changes were observed in stem villi myofibroblasts; these cells had ultrastructural characteristics of fibrocytes with dystrophic changes, (e.g., glycogen granules due to metachoromacy phenomenon, becoming pink after staining with methylene blue).

CD117 and CD34 expression was negative in stem and intermediate villi stroma in the EPE placenta samples (Fig. 3E,F and 4E,F and Table 1). Concentrically located myofibroblasts were found in the vascular walls of stem villi in LPE, and weak CD34 and CD117 staining was only observed in single fields of view. The staining of these samples was predominantly CD34- and CD117-negative (Figs 3G,H and 4G,H). The immunohistochemical study with an antibody for vimentin, a marker for mesenchymal cells, revealed positive staining (++−+++) of the myofibroblasts and smooth muscle cells located in the stem villi stroma (Table 2).

SMA expression in some areas of stem villi was weaker in the EPE placental samples than in the control group (corresponding to the gestational age), though there was negative staining in other areas. In LPE, there were SMA-positive smooth muscle cells with dystrophic changes and irregular expression in stem villi. SMA expression was irregular and less in stem villi smooth muscle cells in patients with LPE than in patients from the control group with corresponding gestational ages.

Immunohistochemical study staining of the stem villi stroma with primary antibodies to TMEM16a were negative in the EPE cases, and only were staining was observed in single cells among the LPE cases (Table 2*)*.

## Discussion

In 1893, neurophysiologist S.R. Cajal described cells in the wall of the small intestine, which subsequently were named after him as interstitial Cajal cells (ICC). TCs interact with one another and other cells via long prolongations and establish a three-dimensional network around blood vessels and nerve endings^20^. This network is one of the key features of TCs^13,14,16,17^. The name of these cells has recently been changed to TCs, and their typical thin, long processes are called Tps. To date, TCs have been found in all gastrointestinal organs^21,22^, urinary and biliary tracts, blood and lymphatic vessel walls^5,21,22^, fallopian tubes, the myometrium^8^, mammary glands^23^ and the placenta^13,14^. To avoid confusion, ICC cells located in organs outside the intestine are denoted ICLCs, though Popescu and Faussone-Pellegrini^16^ called all these cells TCs. Although the placenta is not an innervated organ, we believe that TCs are present. We studied placenta samples during physiological pregnancy and different variants of preeclampsia (PE). We previously detected TCs in placenta villi^18^, which is in agreement with studies by Succiu *et al*.^13,14^. According to the literature, the identification (recognition) of TCs was performed in accordance with the “gold” and “platinum” standards^16,17^. We have shown that there are differences in the ultrastructure of these cells depending on the villi type. Our data showed that all TCs in the placenta have long thin processes with local dilatations, with a few organelles in the perinuclear zone and in the Tp dilatations.

Electron microscopy examination of the term placenta samples showed TCs in different villi types. TC ultrastructural characteristics indicated their heterogeneity and the presence of at least three cell types. All TCs had long thin processes with local dilatations and a small number of organelles in the perinuclear zone. In immature intermediate villi stroma, the TCs formed a network with their Tps that delineated the localization of future blood vessels (stromal channel) (I type). In mature intermediate villi, TCs formed a network under the syncytiotrophoblast basement membrane (II type) and around the villous capillaries (under endothelium) (III type), though in the stem villi vascular walls myofibroblasts were present^18^.

In placental samples from patients with EPE and LPE, the morphological finding on TCs in the stroma of intermediate villi suggest possible fibroblast differentiation based on their larger cytoplasmic volume, the increase in granular endoplasmic reticulum cisternae, and the activation of synthetic processes, as TCs were reduced or absent. In physiological pregnancy, the same morphological changes were observed but only in the single intermediate villi. In contrast, the abovementioned processes were more pronounced in preeclampsia.

Advanced fibrosis of the stroma in intermediate villi in PE had been described previously^24,25^. We previously showed a placenta from a foetus having congenital abnormalities, including ugly mesenchymal stroma cells with thick processes and different sizes and forms. Moreover, TCs were absent^26^.

Dystrophic changes in myofibroblasts in different organs under the hypoxia have been shown in a few studies^27^. TGF-β enhances myofibroblast differentiation, but activation of the FAK signalling pathway leads to the transformation of myofibroblasts into fibrocytes and to the development of fibrosis^28^.

Moreover, the smooth muscle cells and myofibroblasts from stem villi showed dystrophic changes in preeclampsia, including increased cellular vacuolization and accumulation of glycogen granules in the cytoplasm and decreased SMA expression. In patients with LPE, the stem villi, including their stroma, did not correspond to the gestation term (they were predominantly retarded)^24^.

Traditionally, СD117, CD34, and vimentin are known to be TC markers^16,17^. For example, TCs have been described as both CD117-positive and CD117-negative depending on the organ affiliation and localization^16,17^. TCs are probably a heterogeneous cell population^29,30^. Antibodies to TMEM16a (DOG-1) are not TC-specific but are specific for bowel stromal tumours (GIST). Because Vennucchi *et al*. (2013) described different types of TC in the gut, the authors of the manuscript assumed that TMEM16a was specific for TCs^31–33^. TCs in the fallopian tube wall were positive for TMEM16a^34^. TMEM16a is involved in the functioning of calcium-activated chloride channels. We used bowel tissue as a positive control because it has been thoroughly studied as part of the digestive system.

TMEM16a expression is present in TCs in all types of villi (excluding terminal) in the control groups, but displayed more significant expression in myofibroblasts in vascular stem villi walls during full-term pregnancy.

The stem villus originates from an immature intermediate villus, whereby a stromal channel network is reduced, and only a single large vessel remains in the centre of the villus. Stem villi would have dense stroma for the frame of the villous tree. Thus, it is believed that mature intermediate villi stromal channels should gradually disappear after 34 weeks of gestation^4,35^.

T.Cs could potentially mediate the development of vessels and endothelium. The basis for this hypothesis on the mutual influence of TCs and angiogenesis was demonstrated in *in vitro* studies^36^. Zheng Y. *et al*. (2014)^36^ showed that TCs could produce angiogenic factors and cytokines that promote proliferation and the formation of endothelial cells. The study provided the evidence that human lung TCs could produce growth factors, such as VEGF and EGF. Bosco *et al*. also demonstrated that TCs were positive for VEGF^12^. Conditioned culture medium from TCs induced the proliferation of human pulmonary microvascular endothelial cells. Thus, the authors considered that TC might play an important role in angiogenesis^36^.

Recent evidence shows that TC localized in stem villi regulate smooth muscle cell contraction and may be potential regulators of placental villi vascular tonus^5,6,11–14,37^, but our results showed that the cells in the stem villi have features of myofibroblasts.

Impaired intermediate villi TCs have been associated with hypoxia and oxidative stress^2^ and increased concentrations of pro-inflammatory cytokines^38^, which may be factors contributing to the appearance fibrosis in the stroma. This leads to a disturbance in the villous tree blood supply, the progression of foetal hypoxia, development of placental insufficiency, and preeclampsia. The abovementioned disturbances are associated with more severe effects before the 34^th^ week of gestation^39^. Fibrotic stroma is responsible for low bloodstream resistance, impaired compensatory abilities, a tendency to sludge and clot formation.

Normally, the vascular endothelium contains a wide range of antithrombotic and anticoagulant properties. Erythrocyte sludging and microclotting can potentially occur in the damaged endothelium of incompletely formed capillaries^40–42^. It has been found that hypoxia induces the development of new vessels^4,43^; however, these incompletely formed capillaries and vessels are often too narrow and fail to provide adequate blood supply in the placenta. Up to 25% microclotting in intermediate villi has been detected by scanning electron microscopy and previously noted by other authors^44^. Based on NOS expression, the authors assumed that TCs produced NO^17,45–47^. The production of the vasodilator NO in presence of hypoxia is accompanied by the production of the superoxide anion, which leads to the production of the peroxynitrite radical, which is a potent pro-oxidant that is immunohistochemically observed in TCs from PE placentas^12^.

It cannot be ruled out that TCs potentially have other functions. TCs may perform different functions aside from their pacemaker activity, including immunomodulation and functions related to angiogenesis and fibrosis^48–54^. TCs are probably related to stem cells^17,48,49^. The ability of TCs to differentiate into smooth muscle cells has already been described previously^50^.

## Conclusion

In our study, we have found a heterogenetic population of ТСs in the term placenta; these cells differed in their localization, immunophenotype and ultrastructural characteristics. Probably TMEM16a could be used as the marker for identification of TCs. Previously this marker was used for the diagnosis of gastrointestinal stromal tumours (GIST) consisting of TCs^31–33,52^. We suggest the loss and impairment of TCs in preeclampsia (under the influence pathogenic factors, as well as hypoxia). It cannot be ruled out that TCs potentially have other functions aside from their pacemaker activity, including immunomodulation and functions related to angiogenesis and fibrosis^48–50^.

## Methods

We confirm that all methods in this article were performed in accordance with the relevant guidelines and regulations. We confirmed that informed consent was obtained from patients who participated in the study. A statement has been attached.

Placental samples from 37 pregnant patients who had undergone Caesarean section at 25–39 weeks of gestation were examined, including 12 patients diagnosed with early-onset preeclampsia (EPE) and 10 patients with late-onset preeclampsia (LPE). The control group included 15 patients, including 10 patients with physiological full-term pregnancies (late control group) and 5 patients with a Caesarean section at 26–31 weeks not associated with preeclampsia (early control group). Additionally, 15 samples were examined using a ≪Philips CM100≫ electron microscope (Philips/FEI Corporation, Eindhoven, Holland), including 9 samples from the placentas of women with preeclampsia (5 with early-onset preeclampsia and 4 with late-onset preeclampsia) and 6 samples from women with physiological pregnancies.

Criteria for inclusion in the control group meant that placentas were obtained from physiological pregnancies after a Caesarean section that was not performed for the following reasons: (1) a uterine scar, 2) related to obstetrics pathology (e.g., myopia of high degree), or 3) an anatomically narrow pelvis.

Criteria for inclusion in the PE group were as follows: (1) blood pressure >140/90 mm Hg and (2) proteinuria >0.3 g/day^1^.

Criteria for exclusion from both groups including the following: (1) acute and chronic inflammatory diseases, (2) severe extragenital pathology, (3) organ transplantation history, (4) history of oncologic diseases, (5) diabetes, (6) severe foetal pathology, (7) foetal congenital malformations, or (8) spontaneous delivery. All patients signed an informed consent to participate in the study.

After macroscopic examination, tissue fragments from the central zone of each placenta were dissected and fixed in a 10% neutral formalin solution. Paraffin-embedded placental tissue sections (4 µm thick) were used for histological (stained with haematoxylin and eosin) and immunohistochemical examination with primary monoclonal antibodies to CD117 (1:300, clon YR145, Cell Mark, USA), CD34 (ready to use, QBEnd/10; Spring bioscience, USA), vimentin (ready to use, clon SP20, Spring bioscience, USA), SMA (smooth muscle actin) (ready to use, clon 1A4, Dako, Denmark), and the new telocyte marker TMEM16a (DOG-1) (1:100, clon SP31, Abcam, UK)^31–33,52^; the latter was used for the diagnosis of gastrointestinal stromal tumours (GIST) consisting of TCs. Secondary anti-mouse and anti-rabbit antibodies with streptavidin-biotin complexes were also used (SBK KIT DAKO, Denmark).

The positive reaction products were detected by brown staining of the cells. An evaluation was performed using a semiquantitative method with the following grades: (1) 1 point (+) for weak staining, (2) 2 points (++) for moderate staining, and (3) 3 points (+++) for strong staining. As a negative control, the samples from the studied sections underwent a standard immunohistochemical analysis without incubation with primary antibodies. As a positive control, immunohistochemical staining with TMEM16a primary antibodies was performed with the following samples: (1) colon sample of a five-day-old newborn child after a surgical procedure due to the surgical pathology and (2) a small intestine sample taken from an abortion at the 20^th^ week of gestation obtained at an autopsy. For transmission electron microscopy, 1-mm^3^ fragments were taken from deep areas in the placental disk 2–5 minutes after the caesarean section. The material was fixed in 2.5% glutaraldehyde solution and 1% paraformaldehyde solution in 0.1 M phosphate buffer (pH 7.4), and additionally fixed in 1.5% OsO_4_ solution. Soon afterwards, this material was dehydrated and embedded in araldite. Semi-thin sections were stained using the PAS method with additional methylene blue staining. Ultra-thin sections were contrasted with uranyl acetate and plumbum citrate and further examined with a “Philips CM100” electron microscope (Philips/FEI Corporation, Eindhoven, Holland)^55^. Cell size and prolongation thickness were measured by transmission electron microscopy. Statistical data analysis was performed using the “Statistica for Windows v. 8” program package. A difference was considered significant when p ≤ 0.05.

### Ethics

Investigation of the patients’ biological materials was legally confirmed by the patients’ informed consent. The Ethics Commission on biological investigations from the Research Center for Obstetrics, Gynecology and Perinatology from the Ministry of Health of Russia approved the study (protocol of the Commission meeting №6 of 09.06.2016)

All the women provided written informed consent.

This study has been performed within the State Assignment on the topic “Investigation of diagnostic and prognostic roles of molecular-genetic, immunologic and epigenetic factors in preeclampsia development” №116-08-22-1-000-2.
